# Vasoactive Effects of Acute Ergot Exposure in Sheep

**DOI:** 10.3390/toxins13040291

**Published:** 2021-04-20

**Authors:** Rossalin Yonpiam, Jair Gobbet, Ashok Jadhav, Kaushik Desai, Barry Blakley, Ahmad Al-Dissi

**Affiliations:** 1 Department of Veterinary Pathology, Western College of Veterinary Medicine; University of Saskatchewan, Saskatoon, SK S7N 5B4, Canada; rossalin.yonpiam@usask.ca (R.Y.); jag397@mail.usask.ca (J.G.); 2 Department of Pharmacology, College of Medicine University of Saskatchewan, Saskatoon, SK S7N 5E5, Canada; ashok.jadhav@cnl.ca (A.J.); k.desai@usask.ca (K.D.); 3 Department of Veterinary Biomedical Sciences, Western College of Veterinary Medicine; University of Saskatchewan, Saskatoon, SK S7N 5B4, Canada; barry.blakley@usask.ca

**Keywords:** acute ergot exposure, ergot toxicity, sheep, vasoconstriction, adrenergic receptors

## Abstract

Ergotism is a common and increasing problem in Saskatchewan’s livestock. Chronic exposure to low concentrations of ergot alkaloids is known to cause severe arterial vasoconstriction and gangrene through the activation of adrenergic and serotonergic receptors on vascular smooth muscles. The acute vascular effects of a single oral dose with high-level exposure to ergot alkaloids remain unknown and are examined in this study. This study had two main objectives; the first was to evaluate the role of α_1_-adrenergic receptors in mediating the acute vasocontractile response after single-dose exposure in sheep. The second was to examine whether terazosin (TE) could abolish the vascular contractile effects of ergot alkaloids. Twelve adult female sheep were randomly placed into control and exposure groups (*n* = 6/group). Ergot sclerotia were collected and finely ground. The concentrations of six ergot alkaloids (ergocornine, ergocristine, ergocryptine, ergometrine, ergosine, and ergotamine) were determined using HPLC/MS at Prairie Diagnostic Services Inc., (Saskatoon, SK, Canada). Each ewe within the treatment group received a single oral treatment of ground ergot sclerotia at a dose of 600 µg/kg BW (total ergot) while each ewe in the control group received water. Animals were euthanized 12 h after the treatment, and the pedal artery (dorsal metatarsal III artery) from the left hind limb from each animal was carefully dissected and mounted in an isolated tissue bath. The vascular contractile response to phenylephrine (PE) (α_1_-adrenergic agonist) was compared between the two groups before and after TE (α_1_-adrenergic antagonist) treatment. Acute exposure to ergot alkaloids resulted in a 38% increase in vascular sensitivity to PE compared to control (Ctl EC_50_ = 1.74 × 10^−6^ M; Exp EC_50_ = 1.079 × 10^−6^ M, *p* = 0.046). TE treatment resulted in a significant dose-dependent increase in EC_50_ in both exposure and control groups (*p* < 0.05 for all treatments). Surprisingly, TE effect was significantly more pronounced in the ergot exposed group compared to the control group at two of the three concentrations of TE (TE 30 nM, *p* = 0.36; TE 100 nM, *p* < 0.001; TE 300 nM, *p* < 0.001). Similar to chronic exposure, acute exposure to ergot alkaloids results in increased vascular sensitivity to PE. TE is a more potent dose-dependent antagonist for the PE contractile response in sheep exposed to ergot compared to the control group. This study may indicate that the dry gangrene seen in sheep, and likely other species, might be related to the activation of α_1_-adrenergic receptor. This effect may be reversed using TE, especially at early stages of the disease before cell death occurs. This study may also indicate that acute-single dose exposure scenario may be useful in the study of vascular effects of ergot alkaloids.

## 1. Introduction

Ergot poisoning remains an economically important disease affecting a variety of animal species including cattle, sheep, horses, and goats with estimated annual losses of more than a billion dollars within the US [1]. Ergot poisoning is caused by the prolonged consumption of ergot alkaloids which are naturally occurring mycotoxins produced by fungi infecting crops such as triticale, cereals, and grains such as barley, wheat, and durum [2,3,4]. The most widely encountered species of ergot alkaloid producing fungi in Western Canada and Europe are in the family of *Clavicipitaceae* [5,6,7]. This fungal family includes the external spore-producing fungi (*Claviceps* spp.) and endophytic fungi (*Epichloë* spp.). The major species causing agricultural problems in Western Canada is *Claviceps purpura* [5,6]. The active ingredients of ergot alkaloids are confined and concentrated within the sclerotia which are external fungal bodies [8]. Clinical signs of lameness, hoof loss, and dry gangrene of the lower limbs, tail, ear tips, and teats are commonly seen in chronic ergotism and are related to the effect of ergot alkaloids on the vasculature causing severe vasoconstriction [9,10,11]. A complete discussion of the impact of ergot alkaloids on various organs or systems is beyond the scope of this paper. Table 1 provides a brief summary of the main effects reported in the literature (reviewed by Strickland J et al., 2011).

The precise in vivo vasoactive mechanisms of ergot alkaloids have not been determined. However, in vitro tissue bath studies, where normal dissected and isolated arterial rings were exposed to purified ergot alkaloids, have previously shown that the adrenergic and serotonergic receptors on vascular smooth muscles are activated. This is also supported by the fact that the chemical structure, i.e., the ergoline ring, of ergot alkaloids resembles that of physiologic neurotransmitters such as dopamine, norepinephrine, epinephrine, and serotonin, which are known to be vasoactive [4].

It is important to note that despite the rapid metabolism and excretion of ergot alkaloids which occurs within several hours after exposure [12,13], the clinical vascular manifestations of ergot alkaloids are always seen after the prolonged (several weeks to months) consumption of ergot-contaminated plants. While these clinical vascular manifestations could be explained by the repeated exposure to ergot alkaloids, these effects often remain long after the ergot-contaminated feed is removed! Recent evidence suggests that ergot alkaloids may bioaccumulate within the vasculature [14,15]. It is also possible that other unknown vasoactive mechanisms may be involved in mediating these effects.

It is unknown whether acute ergot exposure affects vascular contractility in a similar manner to chronic exposure. If similar effects are found, then acute exposure scenarios may be useful to study the mechanisms of vascular alteration by ergot alkaloids. Many studies have focused on finding an antagonist to counteract the clinical effects of ergotism. Elucidating the mechanisms of vascular contractile response induced by ergot alkaloids may prove useful to identify treatment options for the vascular-related clinical manifestations of ergot poisoning.

This study aimed to examine the role of adrenergic receptors in mediating the vascular effects of ergot alkaloids after an in vivo acute exposure scenario to these alkaloids. Vascular sensitivity to phenylephrine (PE), an α1-adrenergic agonist, was compared between ergot exposed and control groups before and after terazosin (TE), α_1_–adrenergic antagonist, treatment.

We hypothesized that an acute single dose oral exposure to ergot alkaloids results in increased vascular sensitivity (decreased EC50) to PE in the pedal artery; an effect that is mediated through the activation of α_1_-adrenergic receptors. We also hypothesized that the acute vascular effects of oral exposure to ergot alkaloids can be reversed via TE (the α_1_-adrenergic antagonist).

## 2. Results

All animals remained healthy after treatment and did not exhibit any clinical signs during the 12 h period between the administration of ergot alkaloids and euthanasia. No gross or histological changes were seen in either group in the lung, liver, kidneys, heart, spleen, intestines, fat, and pedal arteries. The concentration of ergot alkaloids used to formulate the single oral dose is shown in Table 2.

### 2.1. Phenylephrine Dose Response Curve Compared between Ergot Exposure and Control Groups

**Figure 1:**
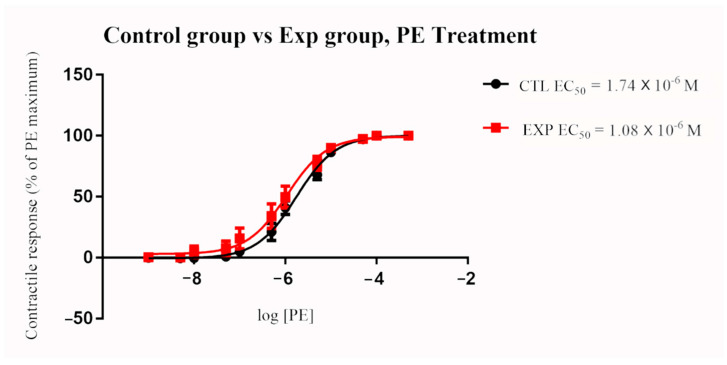
Mean arterial contractile responses to increasing concentration of PE compared between control and ergot exposed group. The pedal artery was collected 12 h after single oral exposure to 600 µg/kg BW (total ergot) or after placebo water treatment (*n* = 6/group). Contractile response data were presented as percentage means ± *SEM* of the maximum contractile effect induced by 1 × 10^−4^ M PE treatment. Ergot exposure resulted in a significant decrease in EC_50_ compared to the control group (*p* < 0.05). EC_50_, the concentration of phenylephrine producing 50% of the maximum contractile response; PE, phenylephrine; BW, body weight; CTL, control group; EXP, exposure group; M, Molar.

In the control group, the PE contractile response was first observed at 1 × 10^−7^ M concentration, and the maximum contractile response recorded at the highest PE concentration (1 × 10^−4^ M) was 22.8 g. The contractile response in the exposure group was first observed at 0.5 × 10^−7^ M while the highest PE concentration yielded a maximum contraction of 18.0 g. Ergot exposure resulted in a significant decrease in EC_50_ compared to the control group (*p* = 0.0462). Comparisons of EC_50_ between the two groups are presented in Figure 1. Details of EC_50_ are for all groups are presented in Table 3.

### 2.2. Effect of Terazosin Treatment on Phenylephrine Dose Response Curve

**Figure 2:**
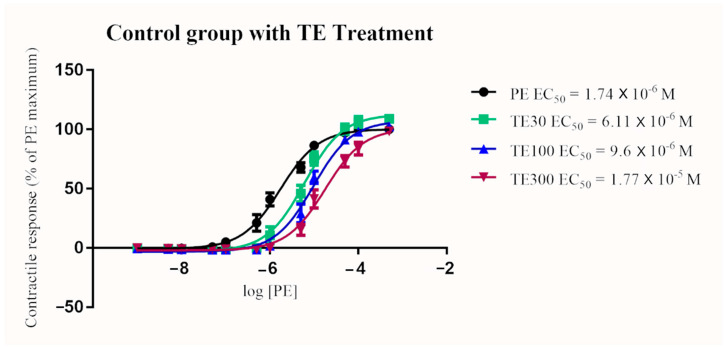
Mean arterial contractile responses to increasing concentration of PE in control animals compared before and after 30, 100, or 300 nM TE treatment (*n* = 6). Contractile response data were presented as percentage means ± *SEM* of the maximum contractile effect induced by 1 × 10^−4^ M PE treatment. TE treatment resulted in a significant dose-dependent increase in EC_50_ compared to PE alone (*p* < 0.0001). EC_50_, the concentration of phenylephrine producing 50% of the maximum contractile response; PE, phenylephrine; TE, terazosin; M, Molar; nM, nanomolar.

**Figure 3:**
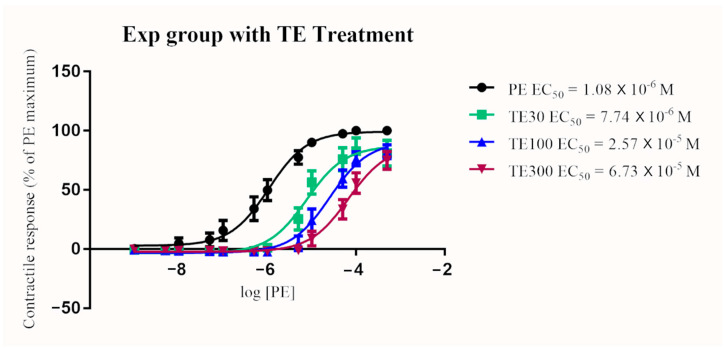
Mean arterial contractile responses to increasing concentration of PE in ergot exposed animals compared before and after 30, 100, or 300 nM TE treatment (*n* = 6). Contractile response data were presented as percentage means ± *SEM* of the maximum contractile effect induced by 1 × 10^−4^ M PE treatment. TE treatment resulted in a significant dose-dependent increase in EC_50_ compared to PE alone (*p* < 0.0001). EC_50_, the concentration of phenylephrine producing 50% of the maximum contractile response; PE, phenylephrine; TE, terazosin; Exp, exposure group; M, Molar; nM, nanomolar.

**Figure 4:**
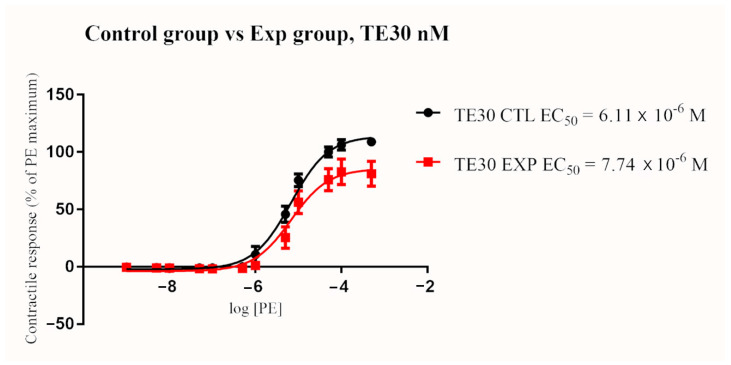
Mean arterial contractile responses to increasing concentration of PE compared between control and ergot exposed groups after TE treatment at 30 nM. EC_50_ was not significantly different between the two groups (*p* = 0.37). Contractile response data were presented as percentage means ± *SEM* of the maximum contractile effect induced by 1 × 10^−4^ M PE treatment. EC_50_, the concentration of phenylephrine producing 50% of the maximum contractile response; PE, phenylephrine; TE, terazosin; CTL, control group; EXP, exposure group; M, Molar; nM, nanomolar.

**Figure 5:**
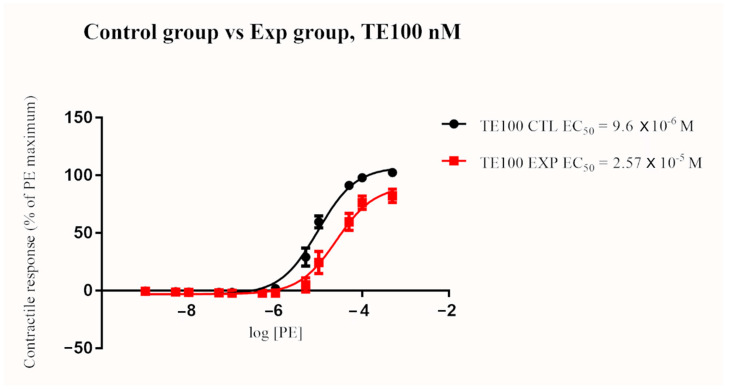
Mean arterial contractile responses to increasing concentration of PE compared between control and exposure after TE treatment at 100 nM. EC_50_ was significantly different between the two groups (*p* < 0.0001). Contractile response data were presented as percentage means ± *SEM* of the maximum contractile effect induced by 1 × 10^−4^ M PE treatment. EC_50_, the concentration of phenylephrine producing 50% of the maximum contractile response; PE, phenylephrine; TE, terazosin; CTL, control group; EXP, exposure group; M, Molar; nM, nanomolar.

**Figure 6:**
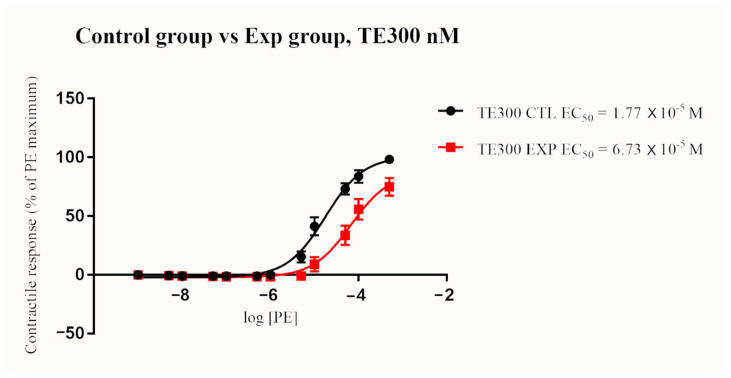
Mean arterial contractile responses to increasing concentration of PE compared between control and exposure after TE treatment at 300 nM. EC_50_ was significantly different between the two groups (*p* < 0.0001). Contractile response data were presented as percentage means ± *SEM* of the maximum contractile effect induced by 1 × 10^−4^ M PE treatment. EC_50_, the concentration of phenylephrine producing 50% of the maximum contractile response; PE, phenylephrine; TE, terazosin; CTL, control group; EXP, exposure group; M, Molar; nM, nanomolar.

In the control group, TE treatment resulted in a significant and dose-dependent increase in EC_50_ (*p* < 0.0001 for all concentrations; 30, 100, and 300 nM). Similarly, EC_50_ significantly increased in a dose-dependent manner in the exposure group after terazosin treatment (*p* < 0.0001 for all concentration; 30, 100, and 300 nM) (Figure 2 and Figure 3). The blocking effect of TE was greater in the exposure group when compared to the control group when given at 100 nM and 300 nM (*p* < 0.0001). A similar trend of increasing EC_50_ in the exposure group compared to the control group after the 30 nM TE treatment was seen, but the difference was not statistically significant (*p* = 0.076) (Figure 4, Figure 5 and Figure 6). (See Table 3 for details).

## 3. Discussion

The biological effects of ergot alkaloids on livestock are known to be diverse. This diversity is not only related to differences in alkaloid concentration and specific alkaloid content in different plants, but also due to their ability to affect multiple biological processes [4,7,16,17]. The ergoline ring system, which is a structure common to ergot alkaloids, is similar to the ring structure of epinephrine, dopamine, and serotonin thus allowing ergot alkaloids to mimic their function. In the vasculature, ergot alkaloids bind with a variety of serotonergic and adrenergic receptors to modify vascular tone [11]. The effects of these alkaloids are known to be diverse with differing potencies among different animal species; however, within a species, the effects are dependent on the animal’s general health, body condition, reproductive status and previous exposure [4,18].

Ergotism in livestock is known to cause dry gangrene due to severe vasoconstriction within peripheral vasculature. Ergotism occurs after the prolonged ingestion of ergot alkaloids. Therefore, previous studies have focused on examining the mechanisms of vasoconstriction following chronic exposure scenarios [19,20,21]. Thus, the vascular effects following acute exposure remain unknown. In this study, we wanted to investigate the role of α_1_-adrenergic receptor activation on vascular contractile response following a single acute high-dose of oral exposure scenario to ergot alkaloids using sheep as a model. Similar to other livestock species, sheep are chronically affected by ergotism and develop dry gangrene after prolonged exposure. We chose to examine the pedal artery due to its peripheral location on the ovine limb.

Ideally, pure individual ergot alkaloids should be used in prolonged feeding trials to precisely examine their vascular effects and the mechanism of these effects. However, because pure ergot alkaloids are very expensive, previous studies often used ergot or endophyte-infected tall fescue. It is often difficult to accurately estimate the individual dose in these studies as the concentration of alkaloids within feed is subject to significant variability due to feed storage conditions and uneven distribution. Alternatively, pure individual alkaloids are often used on dissected arteries to examine their vascular contractile effects in vitro using arterial tissue bath systems. In order to achieve a more defined dosing protocol, we used ground sclerotia in which the concentration of six different ergot alkaloids was determined [16,17]. The dose was adjusted in every animal depending on the body weight to receive a dose of 600 µg/kg BW of total ergot alkaloid content. To the author’s knowledge, no studies have been performed to examine the vascular effects of acute single-dose oral exposure to ergot alkaloids. All previous studies reported vascular effects after repeated low-dose exposure. It is likely that a single low-dose oral exposure will not have a detectable impact on the vasculature. Therefore, to increase the odds of detecting vascular contractile effects after single-dose oral exposure, this study opted to use a high-dose of ergot alkaloids. While the repeated exposure to such a high level is unlikely to occur due to feed refusal, pelleted feed submitted to our diagnostic laboratory from livestock producers for ergot testing occasionally contained similarly high levels.

It is known that the degree of vasoconstriction induced by ergot alkaloids is alkaloid dependent. For example, the vasoconstrictive effect elicited by ergocryptine is 100 times less potent as compared to ergotamine, whereas ergocristine and ergocornine are only 10 times less potent. Ergovaline, the predominant alkaloid in tall fescue grass, is thought to have a similar potency to ergotamine [11,22]. The potency of these alkaloids varies depending on their relative binding affinity to α-adrenergic and serotonergic receptors and their ability to specifically activate them. Most studies examining the vascular effects of ergot alkaloids have focused on studying the serotonergic receptors [19,23,24,25]. However, very few studies examined the activation of α-adrenergic receptors by different alkaloids. For example, the contractile response in the lateral saphenous vein of cattle grazing tall fescue was significantly enhanced compared to control animals by BHT-920, an α_2_-adrenergic agonist, but not by PE (α_1_-adrenergic agonist) [26]. In addition, Schöning et al. reported that ergovaline stimulated α_1_-adrenergic receptors but with low efficacy in rat thoracic aorta [22]. In vivo studies focusing on heart rate and blood pressure changes after exposure to ergot alkaloids also indicate α-adrenergic receptor activation. Bradycardia induced by ergotamine in anesthetized rats was reduced by yohimbine, an α_2_-adrenergic antagonist. In addition, ergotamine treatment reduced the tachycardia induced by electrical stimulation of the spinal cord, and the reduction was similarly blocked after yohimbine treatment [27]. Similarly, in rats, ergotamine has been shown to act as an agonist on α_2_-adrenergic receptors and an antagonist on α_1_-adrenergic receptor [28]. In our study, a significant increase in vascular sensitivity to PE was found in ergot exposed sheep compared to control animals, which might suggest that α_1_-adrenergic receptors mediate that response.

Similar to what we expected, TE decreased the vascular sensitivity to PE in ergot exposed and control sheep due to its antagonistic effects on the α_1_-adrenergic receptor. However, surprisingly, the potency of TE as an α_1_-adrenergic receptor antagonist was significantly enhanced in ergot exposed sheep compared to controls. It has been recently shown that previous exposure to high concentrations of ergot alkaloids may decrease vascular contractility making the vasculature less susceptible to the effects of ergot alkaloids. Klotz et al. examined the contractile response to ergovaline in cattle chronically grazing high and low-endophyte-infected tall fescue [23]. This study demonstrated that the maximum contractile response was significantly higher in steers consuming low-endophyte-infected tall fescue. This is contrary to other studies, which found that the increase in vascular contractile response to ergot alkaloids is dose-dependent [24,29]. It is, thus, possible that the vascular contractile effect of ergot alkaloids is dose-dependent but may become less effective at very high doses. It is possible that the high dose of ergot alkaloids we used resulted in a relatively low contractile response, and also enhanced the blocking effect of TE resulting in a reduced contractile response compared to control tissues. The increased blocking sensitivity of TE in exposed animals may also indicate that the full impact of ergot exposure was not realized due to the short exposure duration.

Alternatively, it is also possible that the effect of the blocker was enhanced in the ergot exposed group due to the unique mixture of alkaloids in the diet. Interestingly, it has been shown that the presence of ergocristine, ergocornine, and ergocryptine together produces adrenergic blockade [30,31,32]. Additionally, Roquebert and Demichel reported that ergocristine acts as an α_1_-adrenergic blocker in rat tail artery [28,33,34]. Ergocristine had the highest concentration in the diet used in this study and may have acted as an antagonist. The enhanced blocking effect of TE in ergot exposed animals may indicate that this blocker may be useful in counteracting the vascular effects of ergot alkaloid exposure.

Several studies have shown that ergot alkaloids interact with serotonin receptors in chronic exposure scenario [19,24,25]. However, Kalkman et al. reported that in rats injected intravenously with ergometrine, the vasoconstrictor response was related to the activation of α_1_- and α_2_-adrenergic receptors, but not serotonergic receptors [35]. It would be interesting to examine the role of serotonergic receptors in mediating arterial contraction after acute exposure to ergot alkaloids. High-level exposure in ruminants can result in nervous signs such as hyperexcitability, hypermetria, and tremors [11,36]. The dose we used was relatively high but was well tolerated by all animals, with none showing clinical signs of illness.

Currently, ergot toxicity is thought to be only related to the prolonged consumption of ergot alkaloids, and it is presumed that a short-term exposure will have no significant clinical effects. However, we show for the first time that even a single oral dose of ergot alkaloids causes a significant increase in sensitivity in arteries supplying distal extremities. This finding is of significance to the livestock industry and regulators, as it may indicate that in cold weather conditions, short-term exposure to ergot alkaloids may result in a significant decrease in blood supply to the extremities, making animals prone to gangrene. It would be interesting to examine whether a similar but lower level exposure scenario would result in a similar response in livestock. In addition, it is also important to examine the effects of a short-term exposure on other systems as it is now presumed that the effects are only seen after chronic exposure. If similar negative effects are seen in other systems, it may indicate the need to lower the allowable limits of ergot alkaloids within feed to reflect the true nature of the negative impact of this disease.

The finding that a blocking effect of TE was more potent in ergot exposed animals may indicate that this drug could be used to treat animals who have been recently exposed to ergot alkaloids. If it is proved to be useful, this drug may significantly reduce the economic impact of ergotism to the livestock industry. It would be interesting to examine whether TE has any impact on other systems affected by this disease.

We recently showed that the S-epimers of ergot alkaloids are vasoactive causing vasoconstriction of bovine dorsal metatarsal arteries in vitro [37]. It is, therefore, possible that the effects seen in this study are related to the combined activity of the R and S epimers and not just the R-epimers.

In summary, this study found that acute high-level exposure to ergot alkaloids results in increased vascular sensitivity to PE and increased blocking effect of TE. Additional studies are immensely needed to examine the role of adrenergic and, serotonergic receptors in other vascular beds in vivo and in vitro after acute exposure.

## 4. Materials and Methods

### 4.1. Animals

All protocols were approved by the Animal Care and Ethics Committee at the University of Saskatchewan (Animal Use Protocol # 20150047, approval date: 31 August 2016). Before the experiment, all animals were weighed and clinically examined with body temperature and heart rate recorded. A blood sample was also collected from each animal, and a complete blood count was performed evaluating red and white blood cell counts as well as platelets count and total plasma protein to ensure that all animals were healthy.

### 4.2. Tissue Collection & Stock Solutions

Twelve healthy adult ewes were randomly assigned into treatment or control groups (*n* = 6/group). Animals were allowed to acclimatize for fourteen days and were fed alfalfa hay and water ad libitum. Ergot alkaloids containing sclerotia were collected, finely ground and the concentrations of six alkaloids (ergocornine, ergocristine, ergocryptine, ergometrine, ergosine, and ergotamine) were determined using HPLC/MS at Prairie Diagnostic Services Inc. (PDS), Saskatoon, SK, Canada (16). Each ewe within the treatment group received a single dose of ground ergot sclerotia at a dose of 600 µg/kg BW (total ergot) dissolved in 50 mL of water via a stomach tube. The concentrations of ergot alkaloids within sclerotia are recorded in Table 1. The control group received water placebo. Twelve hours after treatment, animals were euthanized using a captive bolt and a necropsy was performed. A 15 cm segment of the pedal artery (dorsal metatarsal artery III) was carefully dissected and collected from each animal, soaked in a diluted heparin solution (10 Unit/1 mL) for 2 min and transferred into a container containing modified Krebs–Henseleit buffer solution [in mM: 118 NaCl, 4.7 KCl, 1.2 MgSO_4_, 1.2 KH_2_PO_4_, 22.0 NaHCO_3_, 5.0 glucose and 2.5 CaCl_2_; (Sigma-Aldrich Canada Ltd. Oakville, ON, Canada) (pH 7.4 gassed with 95% O_2_, 5% CO_2_ at 37 °C)] on ice until transport to the laboratory. Immediately upon arrival to the lab, adipose and connective tissue were carefully removed from each arterial segment, which was later sliced into four 3 to 5 mm cross sections. Each arterial section was suspended between the bases of two triangular-shaped wires within an isolated 10 mL tissue bath (Chengdu equipment manufacturing, China) containing modified Krebs–Henseleit buffer solution maintained at the above conditions. Arterial rings were allowed to equilibrate for 1 h under a resting tension of 2 g with the bath solution changed every 15 min. Each day, a fresh stock solution was prepared for phenylephrine (PE) (Sigma-Aldrich Canada Ltd. Oakville, ON, Canada) at a concentration of 1 M, followed by a 10-fold serial dilution to prepare the remaining working solutions. 10 µL were added from each dilution to the 10 mL incubation buffer to obtain the desired final concentration (1 × 10^−9^–1 × 10^−4^ M). Similarly, a fresh initial 1 M stock solution of terazosin (Sigma-Aldrich Canada Ltd. Oakville, ON, Canada) was prepared from which a 10 µM and 100 µM dilutions were made. The desired final concentrations of 30, 100, and 300 nM were prepared by adding 30 µL from the first stock or 10 and 30 µL from the second stock as appropriate. Arterial rings were treated with PE (1 × 10^−4^ M) (Sigma-Aldrich Canada Ltd. Oakville, ON, Canada) to initiate contraction and to confirm tissue viability and responsiveness. The tissues were later washed with incubation buffer until resting tension was achieved.

### 4.3. Contractile Response

Three vascular rings from the pedal artery of each animal were used to assess the PE contractile response before and after the incubation of each ring with a different concentration of TE. Initially, a cumulative concentration-dependent contraction in response to PE was obtained by adding increasing concentrations of PE (1 × 10^−9^ M to 1 × 10^−4^ M). After each PE treatment, arterial rings were allowed to achieve maximum tension which plateaued for 2 min before the next concentration was added. After the last PE treatment, arterial rings were allowed to return to resting tension with buffer replacement occurring every 15 min for 1 h. This was followed by incubating each of the three rings with 30, 100 or 300 nM TE for 20 min after which the cumulative PE contractile response was repeated in each chamber as above. Following completion of the exposure, all rings were exposed to 1 × 10^−4^ M PE to verify their viability.

### 4.4. Data Collection, Analysis and Statistical Analysis

All measured isometric contractile responses were recorded in grams of tension using ‘Chart’ software and Powerlab equipment (AD Instruments Inc., Colorado Springs, CO, USA). For each PE treatment, the maximum tension in grams achieved before the 2 min plateau period was recorded and corrected for a baseline. To minimize variation due to arterial size, each contractile response from an individual ring was normalized to its maximum contractile tension induced by 1 × 10^−4^ M PE treatment.

Contractile response data were presented as percentage means ± *SEM* of the maximum contractile effect induced by 1 × 10^−4^ M PE treatment. For each treatment type, a sigmoidal dose-response curve was plotted using nonlinear regression with variable slope utilizing GraphPad Prism 7 (GraphPad Software Inc., La Jolla, CA, USA), which was later used to calculate potency presented as the concentration producing 50% of the maximum response (EC_50_). Results were presented as the log of the EC_50_ value. Statistical differences in EC_50_ among the different dose-response curves were calculated by the extra sum-of-squares *F*-test where a *p*-value less than 0.05 was considered significant.

## Figures and Tables

**Table 1:** Summary of the main reported effects of ergot alkaloids on different organ or systems in animals (reviewed by Strickland J et al., 2011).

System/Organ Affected	Reported Effect
Gastrointestinal System	- Decreased blood flow to the duodenum and colon - Inhibition of cyclical contractions in the rumen and reticulum - Decreased abomasal motility - Reduced digestibility
Pancreas	- Stimulated insulin secretion - Decreased plasma insulin - Increased activity of protease, trypsin, amylase, and lipase
Neural & Neuroendocrine System	- Endocrine and hypothalamic disruption - Variations in blood concentrations of epinephrine and norepinephrine - Increased nervousness and excitability - Depressed endogenous catecholamine activity - Increased dopamine release - Mixed effects on adrenergic, dopaminergic, and serotoninergic receptors.
Reproductive & Mammary Gland	- Decreased reproductive performance - Decrease pregnancy rate - Decreased prolactin - Decreased milk production - Reduced progesterone - Reduced follicle stimulating and luteinizing hormones - Increased uterine contraction - Decreased number of mature ovarian follicle - Erratic estrous cycles - Decreased dry matter take and nutrition - Decreased neonatal weight & weight gain - reduced sperm motility
Cardiovascular	- Decreased heart rate - Increased vascular tone - Gangrene

**Table 2:** The concentration of six ergot alkaloids determined within ground sclerotia using HPLC/MS *. The total concentration of these alkaloids was used to formulate a single oral dose (600 µg/kg BW) which was administered to each sheep using a stomach tube.

Alkaloid	Concentration (ppb) Dry Weight	Oral Dose (µg/kg BW)
Ergocornine	216,500	26.4
Ergocristine	3,653,000	445.9
Ergocryptine	540,100	65.9
Ergometrine	78,850	9.6
Ergosine	89,570	10.9
Ergotamine	338,300	41.3
Total	4,915,900	600

* The detection limit for each alkaloid was 1.25 ppb. HPLC/MS, high performance liquid chromatography and mass spectrometry; µg/kg BW, microgram per kilogram body weight; ppb, part per billion.

**Table 3:** Phenylephrine (PE) EC_50_ compared between ergot exposed and control sheep (*n* = 6/group) before and after terazosin treatment in dissected pedal arteries using an arterial tissue bath. Ergot exposed sheep received a single oral dose of 600 µg/kg BW total ergot dissolved in a water based on the levels of six ergot alkaloids determined previously. Control sheep received a water placebo treatment. The effect of terazosin was determined using three increasing concentrations of terazosin: 30, 100, and 300 nM. For each treatment type, a sigmoidal dose-response curve was plotted using nonlinear regression which was used to calculate EC_50_. Statistical differences in EC_50_ among the different treatment types were calculated by the extra sum-of-squares *F*-test. A *p*-value less than 0.05 was considered significant.

Tissue Bath Treatment Type	Control EC_50_ and 95% CI	Ergot Exposed EC_50_ and 95% CI	*p*-Value
PE	1.74 × 10^−6^ (1.39 × 10^−6^–2.18 × 10^−6^) ^a,d^	1.08 × 10^−6^ (7.4 × 10^−7^– 1.57 × 10^−6^) ^a,g^	*p* < 0.05
PE + TE (30 nM)	6.11 × 10^−6^ (4.78 × 10^−6^–7.8 × 10^−6^) ^d,e^	7.74 × 10^−6^ (4.63 × 10^−6^–1.3 × 10^−5^) ^g,h^	*p* = 0.37
PE + TE (100 nM)	9.6 × 10^−6^ (7.69 × 10^−6^–1.2 × 10^−5^) ^b,e,f^	2.57 × 10^−5^ (1.7 × 10^−5^–3.9 × 10^−5^) ^b,h,i^	*p* < 0.0001
PE + TE (300 nM)	1.77 × 10^−5^ (1.34 × 10^−5^–2.33 × 10^−5^) ^c,f^	6.73 × 10^−5^ (4.24 × 10^−5^–1.07 × 10^−4^) ^c,i^	*p* < 0.0001

^a–i^ letters with the same superscript are significantly different. EC_50_, the concentration of phenylephrine producing 50% of the maximum contractile response; µg/kg BW, microgram per kilogram body weight; PE, phenylephrine; TE, Terazosin; CI, confidence interval; nM, nanomolar.

## Data Availability

Not applicable.
